# A prognostic CpG score derived from epigenome-wide profiling of tumor tissue was independently associated with colorectal cancer survival

**DOI:** 10.1186/s13148-019-0703-4

**Published:** 2019-07-24

**Authors:** Min Jia, Yan Zhang, Lina Jansen, Viola Walter, Dominic Edelmann, Melanie Gündert, Katrin E. Tagscherer, Wilfried Roth, Melanie Bewerunge-Hudler, Esther Herpel, Matthias Kloor, Alexis Ulrich, Barbara Burwinkel, Hendrik Bläker, Jenny Chang-Claude, Hermann Brenner, Michael Hoffmeister

**Affiliations:** 1 Division of Clinical Epidemiology and Aging Research, German Cancer Research Center (DKFZ), Heidelberg, Germany; 2 Institute of Biostatistics, German Cancer Research Center (DKFZ), Heidelberg, Germany; 3 Division of Molecular Epidemiology, German Cancer Research Center (DKFZ), Heidelberg, Germany; 4 Department of Gynecology and Obstetrics, Molecular Biology of Breast Cancer, University of Heidelberg, Heidelberg, Germany; 5 Institute of Pathology, Heidelberg University Hospital, Heidelberg, Germany; 6 Institute of Pathology, University Medical Center Mainz, Mainz, Germany; 7 Core Facility Genomics & Proteomics, German Cancer Research Center (DKFZ), Heidelberg, Germany; 8 NCT Tissue Bank, National Center for Tumor Diseases (NCT), Heidelberg, Germany; 9 Department of Applied Tumor Biology, Institute of Pathology, Heidelberg University Hospital, Heidelberg, Germany; 10 Department of General, Visceral and Transplantation Surgery, University of Heidelberg, Heidelberg, Germany; 11 Institute of Pathology, Charité University Medicine, Berlin, Germany; 12 Division of Cancer Epidemiology, German Cancer Research Center (DKFZ), Heidelberg, Germany; 13 Division of Preventive Oncology, German Cancer Research Center (DKFZ) and National Center for Tumor Diseases (NCT), Heidelberg, Germany; 14 German Cancer Consortium (DKTK), German Cancer Research Center (DKFZ), Heidelberg, Germany

**Keywords:** Colorectal cancer, Survival, DNA methylation, CpG site, Prognosis

## Abstract

**Background:**

Results of previous studies on the association of the CpG island methylator phenotype (CIMP) with colorectal cancer (CRC) prognosis were inconsistent and mostly based on different CIMP definitions. The current study aimed to comprehensively investigate the associations between DNA methylation on genes previously used to define CIMP status with CRC survival.

**Results:**

Patients with CRC followed up for a median of 5.2 years were divided into a study cohort (*n* = 568) and a validation cohort (*n* = 308). DNA methylation was measured in tumor tissue using the Illumina Infinium HumanMethylation450 BeadChip and restricted to 43 genes used to define CIMP status in previous studies. Cox proportional hazard regression models were used to estimate adjusted hazard ratios (HR) and 95% confidence intervals (CI) of survival after CRC, including adjustment for tumor stage, microsatellite instability, and BRAF mutation status. In the study cohort, ten CpG sites were identified to be associated with CRC survival. Seven of these ten CpG sites were also associated with CRC survival in the validation cohort and were used to construct a prognostic score. CRC patients with a prognostic score of the lowest methylation level showed poorer disease-specific survival compared with patients with the highest methylation level in both the study cohort and the validation cohort (HR = 3.11 and 95% CI = 1.97–4.91, and HR = 3.06 and 95% CI = 1.71–5.45, respectively).

**Conclusions:**

A CpG panel consisting of seven CpG sites was found to be strongly associated with CRC survival, independent from important clinical factors and mutations associated with CIMP. Further studies are required to confirm these findings.

**Electronic supplementary material:**

The online version of this article (10.1186/s13148-019-0703-4) contains supplementary material, which is available to authorized users.

## Background

Colorectal cancer (CRC) is one of the most common cancers worldwide with a 5-year survival rate of more than 60% [1]. Survival after CRC is largely dependent on the disease stage at diagnosis, but there is accumulating evidence that somatic mutations and epigenetic changes play a significant role in the progression of CRC and already guide clinical decision-making [2, 3]. As an important regulation approach in epigenetics, aberrant DNA methylation was extensively observed in various cancers including CRC. High-level methylation of CpG islands in the promoter region (CpG island methylator phenotype (CIMP)) can induce the silencing of the tumor-suppressor genes, which can stimulate tumor onset and progression [4–6]. Colorectal tumors showing such methylation were consistently associated with patient and tumor characteristics, such as older age, proximal location, and microsatellite instability (MSI-H) [7]. However, studies on the association of CIMP status and survival after CRC were inconsistent [8, 9]. In addition to methodological issues in some of the studies, studies mostly used different marker sets to define CIMP, which likely contributed to the inconsistency of results [10, 11].

In previous studies, genes used to define CIMP status were either selected because they were cancer suppressor genes silenced by methylation in the promoter region or because they were differentially methylated in tumor tissue and normal tissue [5, 7, 12]. Although high methylation of CpG islands in the promoter region is presumed to silence these genes, other aberrant methylations on these genes may also influence their activity. Meanwhile, high-throughput assays are available to comprehensively assess methylation of CpGs on CIMP-related genes [13, 14]. We therefore aimed to investigate the association between methylation of CpG sites on genes previously used to define CIMP status with CRC survival.

## Results

### Patient cohort

Of 702 patients in the study cohort and 366 patients in the validation cohort, 134 patients and 58 patients, respectively, were excluded because of missing information on important covariates (chemotherapy, MSI status, or BRAF mutation status). Finally, 568 patients in the study cohort and 308 patients in the validation cohort were included in the analyses. The median follow-up time was 5.2 years in both cohorts. The characteristics of the patients included in the study cohort and validation cohort were very similar. Slight differences were observed in the distribution of the education level, alcohol consumption, lymph node count, chemotherapy frequency, and KRAS mutation (Additional file 1: Table S1).

### Selection of CIMP-related genes

**Fig. 1:**
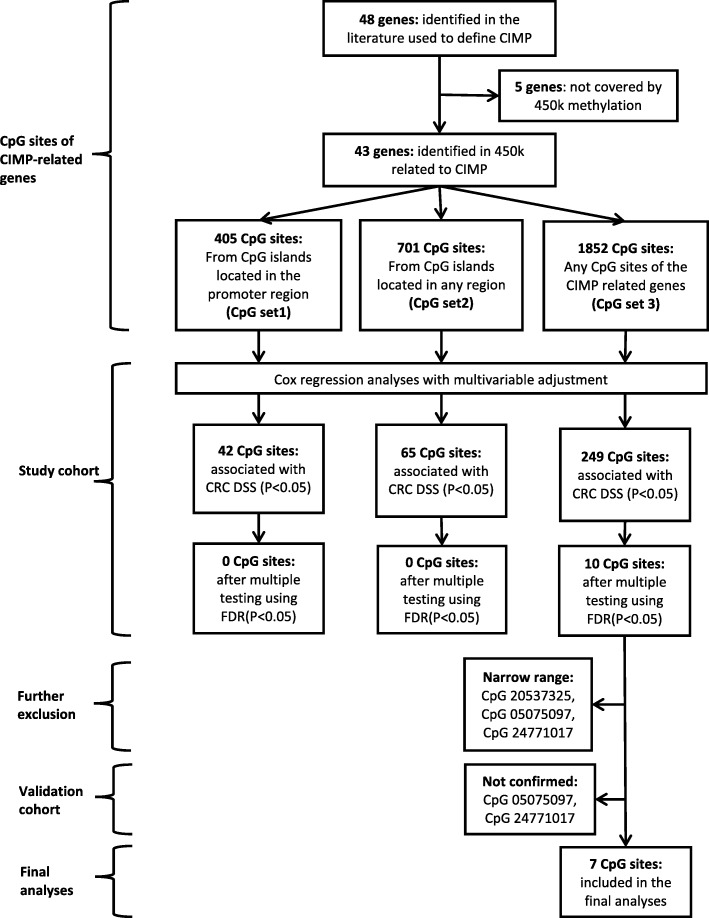
Flow chart of CIMP-related gene selection and identification of prognostic CpG sites associated with disease-specific survival. Cox regression analyses adjusted for age, sex, tumor location, tumor stage, chemotherapy, BRAF mutation, and microsatellite instability

The literature search yielded 48 genes that were used to define CIMP in previous studies [15, 16]. Five of these 48 genes were not covered by the 450k methylation array but were also rarely used for the definition of CIMP (Additional file 1: Table S2). Within the 43 CIMP genes investigated in this study, 405 CpGs were located in CpG islands of the promoter region (CpG set 1), 701 CpGs were located in CpG islands of any region (CpG set 2), and 1852 CpGs were located anywhere on the genes (CpG set 3) (Fig. 1).

### Identification of prognostic CpGs

In adjusted analyses, 42 CpGs from CpG set 1 and 65 CpGs from CpG set 2 were found to be associated with disease-specific survival (DSS) (*p* value<0.05). However, after correction for multiple testing, none of these CpG sites was associated with DSS anymore. By further extension to CpGs anywhere on the genes (CpG set 3), 10 of the 249 associated CpG sites showed significant associations with DSS after correction for multiple testing.

Three of the 10 CpGs had *β* values with a range from the 10th to the 90th percentile of less than 0.1 which was considered too narrow to define distinct groups (cg05075097, cg24771017, and cg20537325). Two of the three CpGs were also not associated with DSS in the validation cohort (cg05075097 and cg24771017). Therefore, seven CpGs (cg16935707, cg05481217, cg08044454, cg01552551, cg24311416, cg02425108, and cg15659052) confirmed in the validation cohort were included in the final analyses (Fig. 1, Additional file 1: Table S3).

### Associations of identified CpG sites with survival

**Fig. 2:**
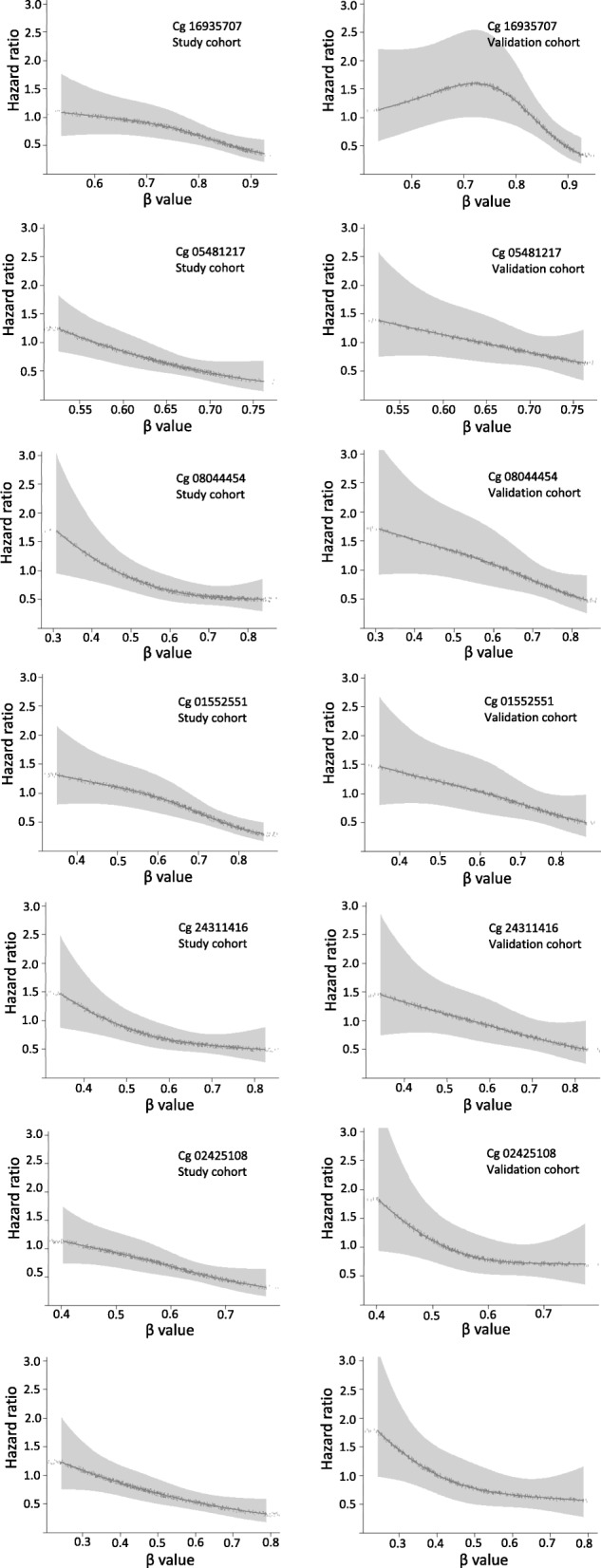
Dose-response association of methylation levels and disease-specific survival in the study cohort and the validation cohort. Restricted cubic splines analyses with adjustment for age, sex, tumor location, tumor stage, chemotherapy, BRAF, mutation and microsatellite instability

All seven CpGs showed similar associations with DSS in the study cohort and the validation cohort (Fig. 2, Additional file 1: Figure S1). Compared to tertile group 3, tertile group 1 (lowest methylation) showed significantly poorer survival for all the seven CpGs in the study cohort. Very similar results were observed in the validation cohort, except for cg02425108, where the association was somewhat weaker (Table 1).

**Table 1:** Association of methylation at the seven identified CpG sites with disease-specific survival in the study cohort and the validation cohort

CpG sites	Methylation level	Study cohort				Validation cohort			
		*N*	Deaths (%)	HR (95% CI)^a^	*p* value of trend	*N*	Deaths (%)	HR (95% CI)^a^	*p* value of trend
cg16935707	*β* value^b^	568		1.43 (1.22–1.67)	< 0.0001	308		1.33 (1.09–1.61)	0.0046
	Tertile 3	193	45 (23)	Ref.		125	27 (22)	Ref.	
	Tertile 2 (0.84342)	182	38 (21)	1.53 (0.98–2.39)		99	25 (25)	1.95 (1.08–3.52)	
	Tertile 1 (0.72736)	193	53 (27)	2.22 (1.46–3.36)	0.0002	84	30 (36)	2.12 (1.20–3.74)	0.0095
cg05481217	*β* value^b^	568		1.43 (1.22–1.67)	< 0.0001	308		1.30 (1.06–1.60)	0.0115
	Tertile 3	187	36 (19)	Ref.		173	42 (24)	Ref.	
	Tertile 2 (0.66890)	183	36 (20)	1.29 (0.79–2.08)		78	17 (22)	1.27 (0.71–2.27)	
	Tertile 1 (0.61265)	198	64 (32)	2.23 (1.46–3.42)	0.0002	57	23 (40)	2.02 (1.18–3.45)	0.0125
cg08044454	*β* value^b^	568		1.44 (1.22–1.69)	< 0.0001	308		1.39 (1.14–1.70)	0.0014
	Tertile 3	195	40 (21)	Ref.		110	20 (18)	Ref.	
	Tertile 2 (0.71507)	195	41 (21)	1.25 (0.80–1.97)		98	28 (29)	1.87 (1.03–3.42)	
	Tertile 1 (0.57145)	178	55 (31)	2.23 (1.44–3.46)	0.0003	100	34 (34)	2.48 (1.39–4.44)	0.0021
cg01552551	*β* value^b^	568		1.43 (1.22–1.68)	< 0.0001	308		1.28 (1.04–1.58)	0.0215
	Tertile 3	189	28 (15)	Ref.		104	25 (24)	Ref.	
	Tertile 2 (0.72226)	189	49 (26)	1.94 (1.21–3.10)		95	22 (23)	1.38 (0.76–2.51)	
	Tertile 1 (0.59834)	190	59 (31)	2.50 (1.58–3.95)	< 0.0001	109	35 (32)	2.17 (1.24–3.79)	0.0065
cg24311416	*β* value^b^	568		1.43 (1.21–1.71)	< 0.0001	308		1.32 (1.04–1.66)	0.0209
	Tertile 3	190	40 (21)	Ref.		118	27 (23)	Ref.	
	Tertile 2 (0.65694)	185	38 (21)	1.03 (0.64–1.64)		90	23 (26)	1.06 (0.59–1.89)	
	Tertile 1 (0.53430)	193	58 (30)	2.20 (1.45–3.35)	0.0002	100	32 (32)	2.00 (1.17–3.43)	0.0135
cg02425108	*β* value^b^	568		1.40 (1.19–1.65)	< 0.0001	308		1.32 (1.04–1.66)	0.0200
	Tertile 3	209	35 (17)	Ref.		104	27 (26)	Ref.	
	Tertile 2 (0.62299)	175	38 (22)	1.78 (1.12–2.85)		107	28 (26)	1.32 (0.75–2.31)	
	Tertile 1 (0.52523)	184	63 (34)	2.44 (1.59–3.74)	< 0.0001	97	27 (28)	1.68 (0.95–2.96)	0.0727
cg15659052	*β* value^b^	568		1.38 (1.17–1.63)	0.0001	308		1.37 (1.09–1.71)	0.0062
	Tertile 3	183	30 (16)	Ref.		113	25 (22)	Ref.	
	Tertile 2 (0.53788)	193	38 (20)	2.12 (1.28–3.51)		92	17 (18)	1.55 (0.80–2.99)	
	Tertile 1 (0.40182)	192	68 (35)	2.34 (1.50–3.67)	0.0002	103	40 (39)	1.99 (1.16–3.34)	0.0118

*Abbreviations*: *HR* hazard ratio, *CI* confidence interval

^a^Cox regression analyses adjusted for age, sex, tumor stage, tumor location, chemotherapy, MSI status, and BRAF mutation status

^b^HR calculated for per unit decrease of the *Z* score of the *β* values

### Associations of the prognostic methylation score with survival

The coefficient values for the calculation of the coefficient score were − 1.24524 for cg16935707, − 1.32717 for cg05481217, − 1.23953 for cg08044454, − 1.15191 for cg01552551, − 0.62350 for cg24311416, − 0.53487 for cg02425108, and − 0.17845 for cg15659052. Using this score based on methylation at seven CpG sites, low methylation was strongly associated with poorer DSS compared with high methylation in the study cohort (HR = 3.16; 95% CI = 2.08–4.80) and in the validation cohort (HR = 2.75; 95% CI = 1.56–4.85) (Table 2). Patients with intermediate methylation scores also showed poorer DSS compared to patients with high methylation scores; however, the trend was not statistically significant.

**Table 2:** Association of the coefficient score with disease-specific survival in the study cohort and the validation cohort

Patient groups	Subgroups	Tertile groups (cutoff points)	Study cohort				Validation cohort			
			*N*	Deaths (%)	HR (95% CI)^a^	*p* value of trend	*N*	Deaths (%)	HR (95% CI)^a^	*p* value of trend
All patients		Tertile 3	195	36 (18)	Ref.		124	25 (20)	Ref.	
		Tertile 2 (− 4.32247)	188	31 (16)	1.31 (0.80–2.15)		89	22 (25)	1.66 (0.91–3.05)	
		Tertile 1 (− 3.92950)	185	69 (37)	3.16 (2.08–4.80)	< 0.0001	95	35 (37)	2.75 (1.56–4.85)	0.0004
Microsatellite instability	MSS	Tertile 3	175	35 (20)	Ref.		114	25 (22)	Ref.	
		Tertile 2 (− 4.32247)	161	29 (18)	1.31 (0.79–2.17)		79	21 (27)	1.63 (0.88–3.01)	
		Tertile 1 (− 3.92950)	167	66 (40)	3.11 (2.04–4.76)	< 0.0001	89	33 (37)	2.65 (1.49–4.72)	0.0009
	MSI-H	Tertile 3	20	1 (5)	Ref.		10	0 (0)	Ref.	
		Tertile 2 (− 4.32247)	27	2 (7)	NC		10	1 (10)	NC	
		Tertile 1 (− 3.92950)	18	3 (17)	NC	NC	6	2 (33)	NC	NC
BRAF mutation	Negative	Tertile 3	185	34 (18)	Ref.		115	22 (19)	Ref.	
		Tertile 2 (− 4.32247)	171	28 (16)	1.23 (0.74–2.07)		81	19 (23)	1.53 (0.81–2.91)	
		Tertile 1 (− 3.92950)	166	61 (37)	3.24 (2.09–5.02)	< 0.0001	93	34 (37)	2.72 (1.53–4.85)	0.0007
	Positive	Tertile 3	10	2 (20)	Ref.		9	3 (33)	Ref.	
		Tertile 2 (− 4.32247)	17	3 (18)	NC		8	3 (38)	NC	
		Tertile 1 (− 3.92950)	19	8 (42)	NC	NC	2	1 (50)	NC	NC
Tumor location	Proximal colon	Tertile 3	59	5 (8)	Ref.		40	10 (25)	Ref.	
		Tertile 2 (− 4.32247)	80	11 (14)	2.60 (0.87–7.84)		27	6 (22)	0.99 (0.31–3.13)	
		Tertile 1 (− 3.92950)	71	25 (35)	6.92 (2.46–19.4)	< 0.0001	41	14 (34)	2.05 (0.79–5.34)	0.1176
	Distal colon	Tertile 3	71	19 (27)	Ref.		41	5 (12)	Ref.	
		Tertile 2 (− 4.32247)	49	5 (10)	0.73 (0.25–2.14)		26	8 (31)	1.76 (0.51–6.09)	
		Tertile 1 (− 3.92950)	56	20 (36)	2.03 (1.05–3.94)	0.0477	21	8 (38)	6.00 (1.61–22.4)	0.0098
	Rectum proximal	Tertile 3	65	12 (18)	Ref.		43	10 (23)	Ref.	
		Tertile 2 (− 4.32247)	59	15 (25)	1.36 (0.62–3.01)		36	8 (22)	3.48 (0.97–12.4)	
		Tertile 1 (− 3.92950)	58	24 (41)	3.59 (1.64–7.87)	0.0012	33	13 (39)	4.02 (1.38–11.7)	0.0129

*Abbreviations*: *CI* confidence interval, *HR* hazard ratio, *NC* not calculated (< 5 patients)

^a^Cox regression adjusted for age, sex, tumor stage, tumor location, chemotherapy, MSI status, and BRAF mutation status

**Fig. 3:**
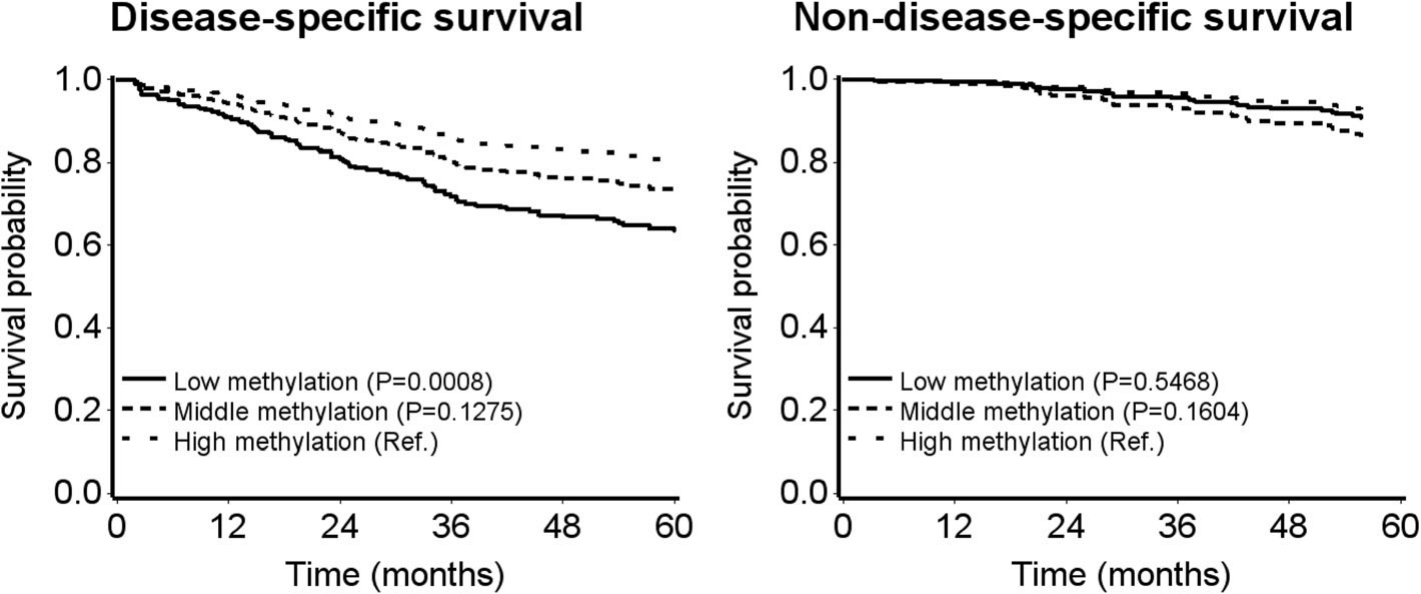
Direct survival curves of the coefficient score with disease-specific survival and non-disease-specific survival in the validation cohort (adjusted for age, sex, tumor location, tumor stage, chemotherapy, BRAF mutation, and microsatellite instability)

Unadjusted Kaplan–Meier curves of the association of the prognostic score with DSS and non-DSS showed poorer survival in the lowest tertile group only (survival rates after 36 months were 68% and 87% for patients in the lowest and highest tertile of the prognostic score, respectively; Additional file 1: Figure S2). Using direct adjusted survival curves, the middle and lowest tertile score were increasingly strongly associated with poorer DSS, whereas no association was found for CRC patients who died of other causes (Fig. 3). In addition, by adding our CpG panel to the model constructed by the clinical and molecular factors, the AIC decreased from 1461 to 1433 in the study cohort and from 823 to 812 in the validation cohort. As most of the CRC patients died of CRC, the association of the methylation panel with overall survival was similar with the association for DSS (data not shown).

In subgroup analyses stratified by tumor location, MSI status, and BRAF mutation status, similar results were observed for the association between the prognostic scores and DSS (Table 2). The prognostic score was not related to clinical and tumor characteristics of the CRC patients such as age, sex, education level, family history of CRC, lifetime regular active smoking status, tumor location, cancer stage, lymph node count, chemotherapy, KRAS mutation, BRAF mutation, and MSI status in both study and validation cohort. Only for chemotherapy heterogeneity was observed in the study cohort due to dissimilar distribution in the tertile 3 group. However, this was not observed in the validation cohort (Additional file 1: Table S4).

## Discussion

In this population-based patient cohort study, we identified seven CpG sites on 43 genes previously used to define CIMP status in the published literature that were independently associated with DSS. At each of the seven CpG sites, lower methylation levels were strongly associated with poorer survival after CRC. The associations observed in the study cohort and the validation cohort were very similar, suggesting that results might be reproducible in other cohorts, too. By incorporating the seven CpGs into a prognostic CpG score we constructed a potential prediction panel for CRC prognosis.

As one of the most widely investigated features of CRC, CIMP was inconsistently associated with poorer outcome in previous studies and the CIMP definitions used were largely different [8, 17]. Mostly, studies focused on CpG sites in the promoter region, where hypermethylation is the aberrant type in cancer cells. In the present study, all seven identified CpGs were selected from CIMP-related genes which we assumed was a more effective approach compared to an epigenome-wide analysis. Recently, a genome-wide methylation study reported a prognostic classifier for non-metastatic CRC consisting of 20 CpGs which also showed poorer survival with lower levels of methylation at the associated CpG sites [18]. The identified CpGs in our study did not overlap, and our score showed a slightly stronger prognostic effect. Among the seven prognostic CpGs identified, cg16935707, cg05481217, and cg24311416 are located in the gene bodies, cg08044454 and cg15659052 are located in the 5′UTR region, cg02425108 is located in the 3′UTR region, and only cg01552551 is located in the S-shore of a CpG island which belongs to the promoter region. At all seven CpG sites, lower methylation instead of higher methylation was associated with poorer survival. Although the molecular mechanism of the association between hypomethylation of CpGs and poorer CRC survival is not known, methylation in the gene body, where hypomethylation is the aberrant type, was found to contribute to cancer-causing somatic and germline mutations in previous studies [19, 20]. In a study on primary glioblastoma, Nagarajan et al. found recurring gene body promoter hypomethylation events which may alter the transcriptional landscape of glioblastoma through the activation of a limited number of normally silenced promoters within gene bodies, in at least one case leading to expression of an oncogenic protein [21].

The seven identified CpGs are located on three different genes: ELMO1 (cg05481217, cg08044454, cg01552552, and cg15659052), CADM1 (cg16935707 and cg24311416), and ADAMTS1 (cg02425108). ELMO1 (engulfment and cell motility 1) together with Dock180 as a complex plays an important role in promoting cancer cell invasion which has been shown for gliomas, breast cancer, and ovarian cancer [22–24]. Methylation markers on ELMO1 were reported to be associated with cancers such as oropharyngeal squamous cell carcinoma and glioblastoma. One other smaller study which adjusted for mucinous type and location only investigated methylation in ELMO1 along with other epigenetic markers in a panel and found worse prognosis in KRAS mutated cancers [25–27]. Cell adhesion molecule 1 (CADM1/TSLC1) is a putative tumor suppressor involved in cell adhesion, proliferation, and apoptosis [28, 29]. The epigenetic inactivation of CADM1/TSLC1 was found as a frequent alteration in the development of CRC and correlates with late stages of the disease [30]. ADAMTS1 (a disintegrin and metalloproteinase with thrombospondin type 1 motifs) is an extracellular matrix metalloproteinase with protease activity and antiangiogenic activity [31]. ADAMTS1 was identified as an epigenetically deregulated gene in CRC, and it was suggested that ADAMTS1 could play an important role in tumor growth and metastasis [32]. All three genes involved are tumor suppressor genes that seem to have effects on tumor growth and metastasis. The alteration of methylation of these genes may play an important role in tumor progression and in the prognosis of CRC patients.

To our knowledge, this is the first study that comprehensively investigates the association between methylation of CpG sites on CIMP-related genes and CRC prognosis in a large patient cohort which is not restricted to specific gene regions. Besides DNA methylation, other molecular pathological features such as MSI, BRAF mutation, and single-nucleotide polymorphisms (SNPs) were frequently investigated for their prognostic value in CRC. For example, MSI-H was found to be a marker of better prognosis among CRC patients in a meta-analysis (OR = 0.58; 95% CI 0.47–0.72), and patients with BRAF mutation were found to have significantly worse progression-free survival compared with BRAF wild-type patients (HR = 1.33; 95% CI 1.12–1.57) [33, 34]. However, as the prognostic values of MSI and BRAF mutation seem to be affected by each other, it was suggested that these markers should be considered jointly [35]. A genome-wide analysis of SNPs illustrated that several SNPs were statistically associated with poorer survival of CRC, with the strongest associations noted for rs209489 (HR = 1.8, *p* = 7.6 × 10^−10^). However, this result has not been validated in an independent cohort yet [36]. Compared with these biomarkers, all the seven prognostic CpGs were selected through multivariable Cox regression with adjustment considering tumor stage, MSI status, and BRAF mutation status as confounders. As validation in an independent cohort yielded very similar results, the panel might be a tool for outcome prediction but further validation in large studies is needed. Besides outcome prediction, further studies will be needed to investigate if and to what extent the CpG score is also useful for the prediction of therapy response.

Both the study cohort and validation cohort were derived from a large population-based study including 876 unselected patients with complete information on clinical and molecular characteristics. Despite the adequate size of the cohort population, the statistical power was still limited in many of the subgroup analyses. Also, the prognostic CpGs identified were selected from CIMP-related genes. It is likely that CpGs of other genes could also be associated with CRC survival, and incorporating these additional sites may further improve the prognostic use of our panel. Accordingly, more large studies on the association between methylation of CpG sites and CRC outcomes are needed without restrictions to specific gene regions. Also, although the CpG sites were confirmed and similarly associated in the validation cohort, external validation in another study is still needed to establish the prognostic score.

## Conclusion

In summary, our study found that DNA methylations at seven CpG sites on CIMP-related genes were strongly associated with CRC prognosis independent of cancer stage, MSI status, BRAF mutation status, and other important factors that affect the outcome of CRC. The prognostic score based on methylation levels at these seven CpG sites strongly predicted CRC survival in the study cohort and the validation cohort. If confirmed in other cohorts, this CpG panel could be a novel tool for CRC outcome prediction and may have relevance for clinical decision-making. Still, other large studies are needed to confirm our findings and to elaborate its potential utility.

## Methods

### Study population

Patients with CRC were recruited into the DACHS (Darmkrebs: Chancen der Verhütung durch Screening) study, a large ongoing population-based cohort study on CRC with long-term follow-up [37]. Patients histologically confirmed CRC who were at least 30 years old and physically and mentally able to participate in a personal interview were enrolled in 22 hospitals in the Rhine–Neckar–Odenwald region of southwestern Germany. Sociodemographic and lifestyle information was collected by trained interviewers using a standardized questionnaire during face-to-face interviews. Additionally, discharge letters, pathology reports, and endoscopy reports were collected at baseline. After 3 years, information on the patients’ therapy was requested from the patients’ physicians. After 5 years, survivors were contacted to complete a standardized questionnaire. In addition, at both follow-ups, disease recurrence was assessed. Also, data on vital status were obtained from the population registries and causes of death were verified by death certificates from the health authorities. More details on the study design, data collection, and follow-up have been reported previously [38, 39].

In the present study, only patients recruited between 2003 and 2007 with available information on DNA methylation were included. The study was approved by the ethics committees of the Medical Faculty of the University of Heidelberg and of the Medical Chambers of Baden–Wuerttemberg and Rhineland–Palatinate.

### Tumor sample analyses

DNA was extracted from formalin-fixed, paraffin-embedded (FFPE) tumor samples of the patients under microscopic control of unstained slides and was prepared using the DNeasy tissue kit (Qiagen, Hilden, Germany) [40]. Methylation of tissue DNA was analyzed using the Illumina Human Methylation 450 BeadChip (Illumina, San Diego, CA, USA) following the manufacturer’s instructions. Tumors of patients examined in the pathology institutes in Heilbronn, Ludwigshafen, Mannheim, and Speyer (study cohort) were analyzed more than 1 year apart from tumors of patients examined in the pathology institute in Heidelberg (validation cohort). Methylation signals at CpG sites were converted into *β* values (methylated signal/(unmethylated signal + methylated signal)). *β* values ranged from 0 to 1: 0 represents totally unmethylated and 1 represents totally methylated. In data pre-processing, the following probes were excluded: probes targeting the X and Y chromosomes, probes containing a single-nucleotide polymorphism (dbSNP132 Common) within five base pairs, probes not mapping uniquely to the human reference genome (hg19) allowing for one mismatch, and probes that have failed in more than 10% of the samples based on the detection *p* value (detection *p* value > 0.01). Data was normalized by pre-processing in the R package “minfi” [41].

MSI status was determined using a mononucleotide marker panel (BAT25, BAT26, and CAT25) [42]. KRAS mutation was determined by single-stranded conformational polymorphism technique using the same DNA sample [43]. The expression of BRAF V600E was determined by immunohistochemical analyses in sections of tissue microarray blocks and evaluated by two pathologists independently.

### Statistical analysis

Genes used to define CIMP in previous studies were identified by a literature review [15, 16]. We focused on CIMP genes because CIMP is an established marker in CRC to differentiate between CRC pathways. We assumed that methylation on these genes could be more relevant than on other genes when investigating associations with survival. Also, by focusing on CIMP genes, the number of association tests is lower which increases the chance to find associations even after adjustment for multiple testing.

In a split-sample approach, we used a larger study cohort to investigate associations of CpG sites on CIMP-related genes with DSS and a smaller validation cohort to validate our findings. Hazard ratios (HR) and 95% confidence intervals (95% CI) were calculated by multivariable Cox proportional hazard regression with adjustment for age, sex, tumor stage, tumor location, chemotherapy, MSI status, and BRAF mutation status. In addition, a correction for late entry defined as the potentially delayed time between the date of diagnosis and the date of enrolment was included in the adjusted model, as well as a time-dependent variable of age and chemotherapy. We used three different sets of CpGs on the CIMP genes to test for associations: CpG set 1 consisted of CpGs located in the CpG islands of promoter regions, CpG set 2 included any CpG in CpG islands, and CpG set 3 included any CpG in any region on the genes (Fig. 1). In the study cohort, CpGs were deemed to be associated with CRC prognosis only if they passed the correction for multiple testing by Benjamini–Hochberg (false discovery rates (FDR) < 0.05) and if the *β* value range was not too narrow (≥ 0.1) to ensure reproducibility.

The CpGs associated with DSS in the study cohort were then analyzed in the validation cohort using the same adjusted Cox model. The association between tertiles of *β* values and DSS analyzed in the study cohort was analyzed in the validation cohort using the tertile cutoffs of the study cohort. Dose-response curves of methylation levels at the identified CpGs were plotted to illustrate the association with DSS by restricted cubic spline regression adjusting for the confounders mentioned above. The CpGs confirmed in the validation cohort were used to investigate individual associations and to construct a prognostic score using the coefficient score. A heatmap illustrating the methylation level of the selected CpGs and their association with CRC survival was generated using the R package “pheatmap”.

The coefficient score was calculated by summing the product of the *β* value of each validated CpG with its corresponding coefficient value in the Cox regression. Tertiles of the sum were used to build three groups with tertile 3 representing patients with the highest methylation at the selected CpGs sites, which was used as the reference (among the identified CpGs, lower methylation was associated with higher mortality). The coefficient value and tertile cutoff point identified in the study cohort were used for the analyses in the validation cohort. Direct adjusted survival curves (adjusting for the same covariates) and Kaplan–Meier curves were plotted to illustrate the association of the prognostic score with DSS and non-DSS (deaths due to other causes) over time. The associations of clinical and molecular features with the coefficient score in the study and the validation cohort were analyzed by chi-square test. The Akaike information criterion (AIC) was computed using R, software version 3.5.0. All other statistical analyses were performed with SAS, software version 9.4 (SAS Institute Inc., Cary, NC, USA).

## Additional file

Additional file 1: **Table S1.** Characteristics of patients in the study cohort and the validation cohort. **Table S2.** Genes used to define CIMP status in previous studies that were available on the 450k methylation array. **Table S3.** CpG sites identified in the study cohort that were included or excluded in the final analyses. **Table S4.** Association of the coefficient score with characteristics of colorectal cancer patients in the study cohort and the validation cohort. **Figure S1.** Heatmaps showing the methylation level of the seven CpG sites across all the patients in the study cohort (A) and the validation cohort (B), respectively. **Figure S2.** Unadjusted Kaplan–Meier curves of the association of the coefficient score with disease-specific survival and non-disease-specific survival in the validation cohort. (DOCX 2549 kb)

## Data Availability

The datasets used and/or analyzed during the current study are available from the corresponding author on reasonable request.

## Abbreviations

AIC: Akaike information criterion
CI: Confidence interval
CIMP: CpG island methylator phenotype
CpGs: CpG sites
CRC: Colorectal cancer
DSS: Disease-specific survival
HR: Hazard ratio
MSI: Microsatellite instability
MSI-H: High-level microsatellite instability
