# Supplementary material for: A prognostic CpG score derived from epigenome-wide profiling of tumor tissue was independently associated with colorectal cancer survival
Source: Clin Epigenetics. 2019 Jul 24;11:109. doi: 10.1186/s13148-019-0703-4 (PMC6657180; doi:10.1186/s13148-019-0703-4)
Supplement: Supplementary file 1 — Table S1. Characteristics of patients in the study cohort and the validation cohort. Table S2. Genes used to define CIMP status in previous studies that were available on the 450k methylation array. Table S3. CpG sites identified in the study cohort that were included or excluded in the final analyses. Table S4. Association of the coefficient score with characteristics of colorectal cancer patients in the study cohort and the validation cohort. Figure S1. Heatmaps showing the methylation level of the seven CpG sites across all the patients in the study cohort (A) and the validation cohort (B), respectively. Figure S2. Unadjusted Kaplan–Meier curves of the association of the coefficient score with disease-specific survival and non-disease-specific survival in the validation cohort. (DOCX 2549 kb) [file 13148_2019_703_MOESM1_ESM.docx]

**Table S 1**. Characteristics of patients in the study cohort and the validation cohort.

| Characteristics |  | Study cohort (n=568) | Validation cohort (n=308) | P value for heterogeneity |
| --- | --- | --- | --- | --- |
| Age, n (%) |  |  |  | 0.1316 |
| <=65 y |  | 198 (35) | 124 (40) |  |
| 66-74 y |  | 194 (34) | 86 (28) |  |
| 75+ y |  | 176 (31) | 98 (32) |  |
| Sex, n (%) |  |  |  | 0.1579 |
| Female |  | 268 (47) | 130 (42) |  |
| Male |  | 300 (53) | 178 (58) |  |
| Education, n (%) |  |  |  | 0.0218 |
| Low |  | 380 (67) | 190 (62) |  |
| Medium |  | 117 (21) | 58 (19) |  |
| High |  | 71 (13) | 60 (19) |  |
| Family history of CRC, n (%)^a^ |  | 81 (14) | 47 (15) | 0.6839 |
| Lifetime regular active smoking, n (%)^b^ |  |  |  | 0.5390 |
| None |  | 258 (46) | 125 (41) |  |
| <20 pack-years |  | 190 (34) | 108 (35) |  |
| 20+ pack-years |  | 119 (21) | 73 (24) |  |
| Alcohol consumption, mean (g/day) ^c^ |  | 16.1 | 20.3 | 0.0009 |
| Body mass index, mean (kg/m^2^)^d^ |  | 26.5 | 26.4 | 0.6692 |
| Physical activity, mean (life time METs, hr/week)^e^ |  | 240.7 | 237.2 | 0.6983 |
| Regular use of NSAIDs, n (%)^f^ |  | 147 (26) | 90 (29) | 0.2887 |
| Regular use of statins, n (%)^g^ |  | 61 (11) | 47 (15) | 0.0544 |
| Regular use of hormone replacement therapy, n (%)^h^ |  | 82 (14) | 38 (12) | 0.3927 |
| Tumor location, n (%) |  |  |  | 0.4279 |
| Proximal colon |  | 210 (37) | 108 (35) |  |
| Distal colon |  | 176 (31) | 88 (29) |  |
| Rectum |  | 182 (32) | 112 (36) |  |
| Cancer stage, n (%) |  |  |  | 0.3926 |
| I |  | 105 (18) | 54 (18) |  |
| II |  | 184 (32) | 113 (37) |  |
| III |  | 192 (34) | 89 (29) |  |
| IV |  | 87 (15) | 52 (17) |  |
| Lymph node count, n (%) |  |  |  | 0.0013 |
| <=12 |  | 178 (31) | 82 (27) |  |
| 12-20 |  | 274 (48) | 129 (42) |  |
| 20+ |  | 116 (20) | 97 (31) |  |
| Surgery, n (%) |  | 567 (100) | 308 (100) | 0.4612 |
| Chemotherapy, n (%) |  | 270 (48) | 121 (39) | 0.0190 |
| KRAS mutation, n (%)^i^ |  |  |  | 0.0008 |
| Negative |  | 324 (66) | 224 (77) |  |
| Positive |  | 168 (34) | 66 (23) |  |
| BRAF mutation, n (%) |  |  |  | 0.2981 |
| Negative |  | 522 (92) | 289 (94) |  |
| Positive |  | 46 (8) | 19 (6) |  |
| Microsatellite instability, n (%) |  |  |  |  |
| MSS |  | 503 (89) | 282 (92) | 0.1644 |
| MSI-H |  | 65 (11) | 26 (8) |  |

Missing data for (a) 3 patients of the study set and for 2 patients of the validation set, (b) 1 patient of the study set and for 2 patients of the validation set, (c) 3 patients of the study set and for 4 patients of the validation set, (d) 1 patient of the study set and for 4 patients of the validation set, (e) 15 patients of the study set and for 9 patients of the validation set, (f) 2 patients of the study set and for 1 patient of the validation set, (g) 2 patients of the study set, (h) 1 patient of the study set and for 1 patient of the validation set, (i) 76 patients of the study set and for 18 patients of the validation set.

**Table S 2**. Genes used to define CIMP status in previous studies that were available on the 450k methylation array.

| Gene name^a^ | Covered by 450k |  | Gene name^a^ | Covered by 450k |  | Gene name^a^ | Covered by 450k |  | Gene name^a^ | Covered by 450k |
| --- | --- | --- | --- | --- | --- | --- | --- | --- | --- | --- |
| ADAMTS1 | √ |  | APC | √ |  | BRAF | √ |  | CACNA1G | √ |
| CDH13 | √ |  | CHFR | √ |  | CRABP1 | √ |  | DUSP26 | √ |
| EDIL3 | √ |  | ELMO1 | √ |  | FBN2 | √ |  | GATA5 | √ |
| HAND1 | √ |  | HCAD | √ |  | HIC1 | √ |  | HLTF | √ |
| HRK | √ |  | ID4 | √ |  | IGF2 | √ |  | IGFBP3 | √ |
| KIRREL2 | √ |  | LOX | √ |  | MDR1 | √ |  | MGMT | √ |
| MINT1 | √ |  | MINT2 | √ |  | MINT12 | × |  | MINT17 | × |
| MINT25 | × |  | MINT27 | × |  | MINT31 | × |  | MLH1 | √ |
| Neurog1 | √ |  | P14 | √ |  | P16 | √ |  | RASSF2 | √ |
| RIZ1 | √ |  | RSASF1A | √ |  | RUNX3 | √ |  | SLC13A5 | √ |
| SOCS1 | √ |  | STOX2 | √ |  | THBD | √ |  | TIMP3 | √ |
| TSLC1 | √ |  | TSP1 | √ |  | UCHL1 | √ |  | WRN | √ |

a: UCSC RefGene name.

**Table S 3**. CpG sites identified in the study cohort that were included or excluded in the final analyses.

| CpG site | Association with disease-specific survival^a^ (HR, 95%CI) | | Not confirmed  in validation cohort | 10%--90% range of  β value (distance) | Distance < 0.1 | Finally included |
| --- | --- | --- | --- | --- | --- | --- |
|  | Study cohort | Validation cohort |  |  |  |  |
| cg16935707 | 1.43 (1.22-1.67) | 1.33 (1.09-1.61) |  | 0.612--0.890 (0.278) |  | √ |
| cg05481217 | 1.43 (1.22-1.67) | 1.30 (1.06-1.60) |  | 0.543--0.705 (0.162) |  | √ |
| cg08044454 | 1.44 (1.22-1.69) | 1.39 (1.14-1.70) |  | 0.426--0.790 (0.364) |  | √ |
| cg01552551 | 1.43 (1.22-1.68) | 1.28 (1.04-1.58) |  | 0.451--0.816 (0.365) |  | √ |
| cg20537325 | 1.34 (1.18-1.55) | 1.31 (1.05-1.63) |  | 0.889--0.932 (0.043) | × |  |
| cg24311416 | 1.43 (1.21-1.71) | 1.32 (1.04-1.66) |  | 0.412--0.759 (0.347) |  | √ |
| cg02425108 | 1.40 (1.19-1.65) | 1.32 (1.04-1.66) |  | 0.440--0.709 (0.269) |  | √ |
| cg05075097 | 1.51 (1.23-1.86) | 1.05 (0.83-1.33) | × | 0.053--0.106 (0.053) | × |  |
| cg15659052 | 1.38 (1.17-1.63) | 1.37 (1.09-1.71) |  | 0.293--0.689 (0.396) |  | √ |
| cg24771017 | 1.32 (1.14-1.54) | 1.04 (0.83-1.31) | × | 0.858--0.953 (0.095) | × |  |

a: Cox regression analyses using the z-score of the β value (per unit, high to low) with adjustment for age, sex, tumor stage, tumor location, chemotherapy, MSI status and BRAF mutation status.

**Table S 4**. Association of the coefficient score with characteristics of colorectal cancer patients in the study cohort and the validation cohort.

| Characteristics | Study cohort (n=568) | | | |  | Validation cohort (n=308) | | | |
| --- | --- | --- | --- | --- | --- | --- | --- | --- | --- |
|  | Tertile1 | Tertile2 | Tertile3 | P value |  | Tertile1 | Tertile2 | Tertile3 | P value |
| Age, n (%) |  |  |  |  |  |  |  |  |  |
| <=65 y | 56 (30) | 68 (36) | 74 (38) |  |  | 43 (45) | 39 (44) | 42 (34) |  |
| 66-74 y | 68 (37) | 63 (34) | 63 (32) |  |  | 21 (22) | 25 (28) | 40 (32) |  |
| 75+ y | 61 (33) | 57 (30) | 58 (30) | 0.6112 |  | 31 (33) | 25 (28) | 42 (34) | 0.3208 |
| Sex, n (%) |  |  |  |  |  |  |  |  |  |
| Female | 83 (45) | 100 (53) | 85 (44) |  |  | 39 (41) | 35 (39) | 56 (45) |  |
| Male | 102 (55) | 88 (47) | 110 (56) | 0.1266 |  | 56 (59) | 54 (61) | 68 (55) | 0.6708 |
| Education, n (%) |  |  |  |  |  |  |  |  |  |
| Low | 121 (65) | 134 (71) | 125 (64) |  |  | 54 (57) | 51 (57) | 85 (69) |  |
| Medium | 43 (23) | 35 (19) | 39 (20) |  |  | 19 (20) | 23 (26) | 16 (13) |  |
| High | 21 (11) | 19 (10) | 31 (16) | 0.3188 |  | 22 (23) | 15 (17) | 23 (19) | 0.1198 |
| Family history of CRC, n (%) |  |  |  |  |  |  |  |  |  |
| No | 157 (85) | 158 (84) | 169 (88) |  |  | 79 (83) | 78 (88) | 102 (84) |  |
| Yes | 28 (15) | 29 (16) | 24 (12) | 0.6462 |  | 16 (17) | 11 (12) | 20 (16) | 0.6465 |
| Lifetime regular active smoking, n (%) |  |  |  |  |  |  |  |  |  |
| None | 82 (44) | 87 (47) | 89 (46) |  |  | 32 (34) | 36 (41) | 57 (48) |  |
| <20 pack-years | 58 (31) | 63 (34) | 66 (34) |  |  | 40 (42) | 27 (31) | 38 (32) |  |
| 20+ pack-years | 45 (24) | 36 (19) | 38 (20) | 0.7787 |  | 23 (24) | 25 (28) | 25 (21) | 0.2068 |
| Tumor location, n (%) |  |  |  |  |  |  |  |  |  |
| Proximal colon | 71 (38) | 80 (43) | 59 (30) |  |  | 41 (43) | 27 (30) | 40 (32) |  |
| Distal colon | 56 (30) | 49 (26) | 71 (36) |  |  | 21 (22) | 26 (29) | 41 (33) |  |
| Rectum | 58 (31) | 59 (31) | 65 (33) | 0.1087 |  | 33 (35) | 36 (40) | 43 (35) | 0.2420 |
| Cancer stage, n (%) |  |  |  |  |  |  |  |  |  |
| I | 32 (17) | 42 (22) | 31 (16) |  |  | 15 (16) | 13 (15) | 26 (21) |  |
| II | 58 (31) | 67 (36) | 59 (30) |  |  | 31 (33) | 35 (39) | 47 (38) |  |
| III | 60 (32) | 63 (34) | 69 (35) |  |  | 31 (33) | 28 (31) | 30 (24) |  |
| IV | 35 (19) | 16 (9) | 36 (18) | 0.0604 |  | 18 (19) | 13 (15) | 21 (17) | 0.6494 |
| Lymph node count, n (%) |  |  |  |  |  |  |  |  |  |
| <=12 | 50 (27) | 59 (31) | 69 (35) |  |  | 19 (20) | 24 (27) | 39 (31) |  |
| 12-20 | 90 (49) | 93 (49) | 91 (47) |  |  | 39 (41) | 41 (46) | 49 (40) |  |
| 20+ | 45 (24) | 36 (19) | 35 (18) | 0.3553 |  | 37 (39) | 24 (27) | 36 (29) | 0.2225 |
| Chemotherapy, n (%) |  |  |  |  |  |  |  |  |  |
| No | 95 (51) | 112 (60) | 91 (47) |  |  | 54 (57) | 52 (58) | 81 (65) |  |
| Yes | 90 (49) | 76 (40) | 104 (53) | 0.0382 |  | 41 (43) | 37 (42) | 43 (35) | 0.3874 |
| KRAS mutation, n (%) |  |  |  |  |  |  |  |  |  |
| Negative | 102 (61) | 115 (71) | 107 (66) |  |  | 67 (73) | 72 (86) | 85 (75) |  |
| Positive | 65 (39) | 47 (29) | 56 (34) | 0.1656 |  | 25 (27) | 12 (14) | 29 (25) | 0.0856 |
| BRAF mutation, n (%) |  |  |  |  |  |  |  |  |  |
| Negative | 166 (90) | 171 (91) | 185 (95) |  |  | 93 (98) | 81 (91) | 115 (93) |  |
| Positive | 19 (10) | 17 (9) | 10 (5) | 0.1565 |  | 2 (2) | 8 (9) | 9 (7) | 0.1233 |
| Microsatellite instability, n (%) |  |  |  |  |  |  |  |  |  |
| MSS | 167 (90) | 161 (86) | 175 (90) |  |  | 89 (94) | 79 (89) | 114 (92) |  |
| MSI-H | 18 (10) | 27 (14) | 20 (10) | 0.3031 |  | 6 (6) | 10 (11) | 10 (8) | 0.4777 |


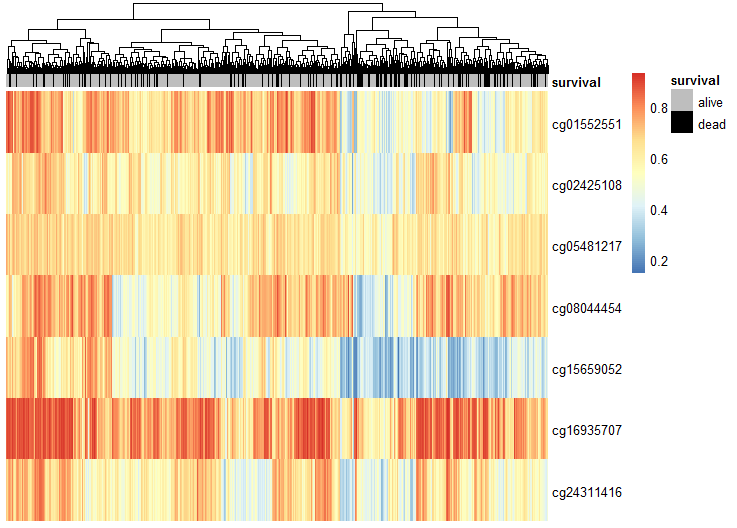

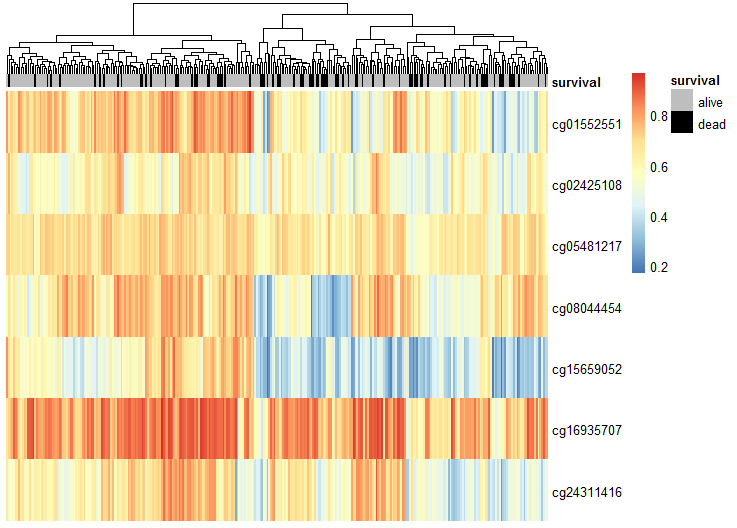


**(A) Study cohort**

**(B) Validation cohort**

**Figure S 1**. Heatmaps showing the methylation level of the seven CpG sites across all the patients in the study cohort (A) and the validation cohort (B), respectively.


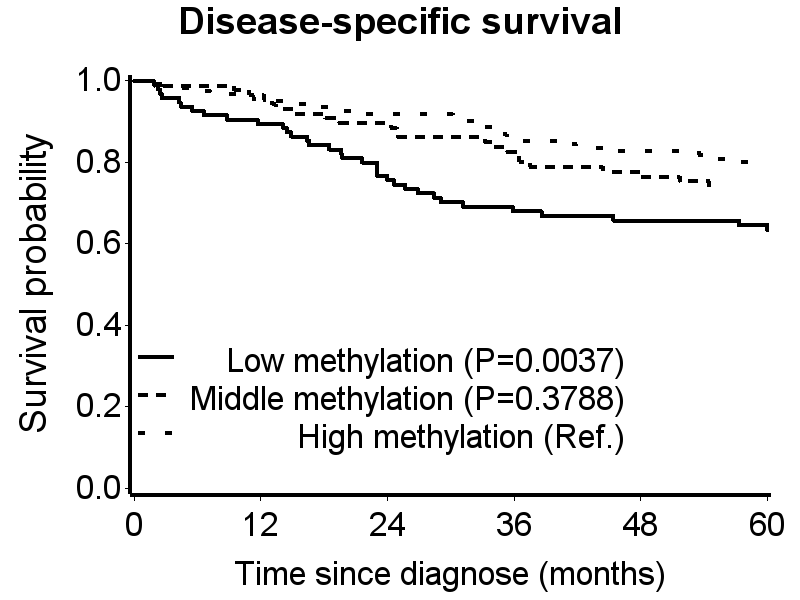

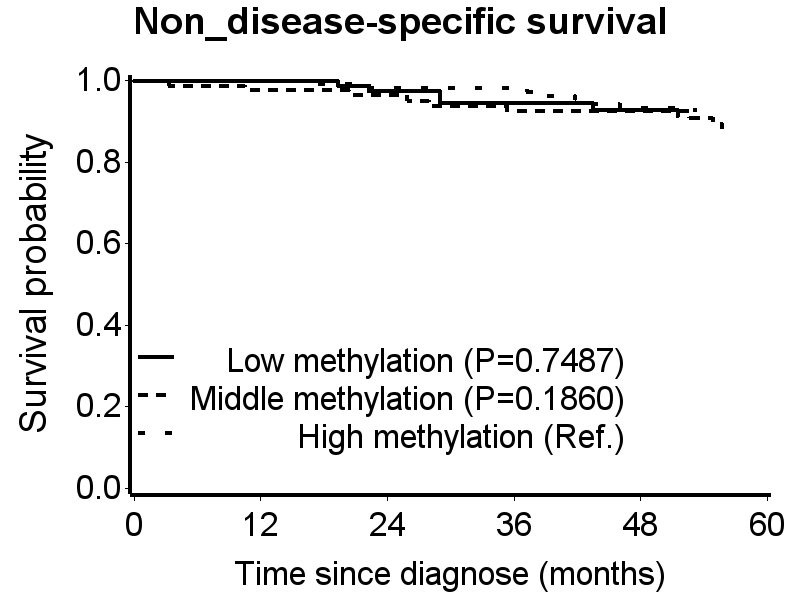


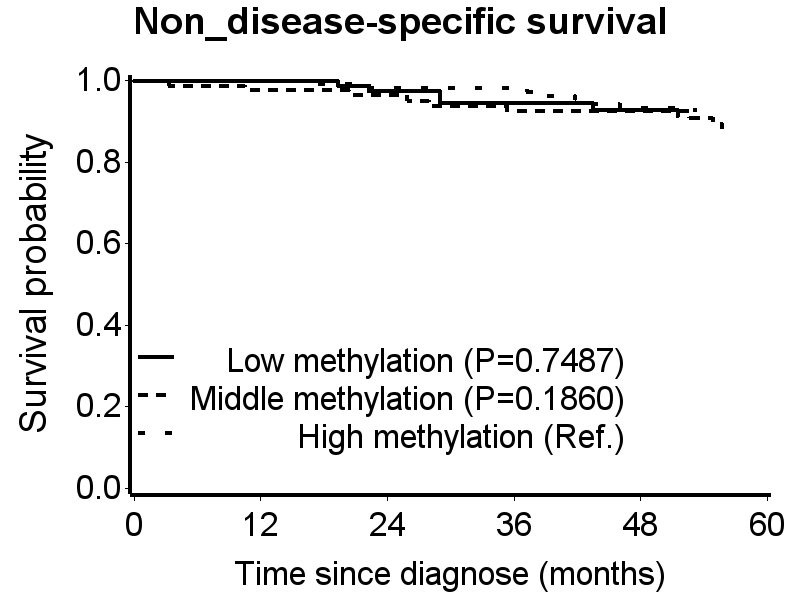

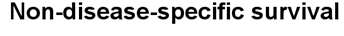


**Figure S 2**. Unadjusted Kaplan-Meier curves of the association of the coefficient score with disease-specific survival and non-disease-specific survival in the validation cohort.
